# Serendipity in medical education

**DOI:** 10.3205/zma001766

**Published:** 2025-06-16

**Authors:** Sigrid Harendza

**Affiliations:** 1 Universitätsklinikum Hamburg-Eppendorf, III. Medizinische Klinik, Hamburg, Germany

## Editorial

If Sir Alexander Fleming had simply thrown away his bacterial cultures destroyed by a fungus as a failed experiment and had not turned his attention to the question of why bacterial growth was inhibited, penicillin would probably not have been discovered at that time [1]. What a happy coincidence to observe something by chance that turns out to be an unexpected discovery! This basic principle of serendipity also includes the willingness to perceive and consciously use the unexpected. Serendipity was also at play when a reduction in leukocytes was observed in the examination of soldiers exposed to mustard gas, which led to the approval of mustard gas as the first chemotherapy a few years later [2]. In postgraduate medical education, serendipity is also described as an important aspect of professional development, especially if you happen to meet the “right” role models and mentors [3], [4], [5]. Similarly, serendipity was identified as a key factor for starting a career in the field of medical education in a qualitative study in 2015 [6]. Knowing serendipity as a principle and being able to work with it, therefore, appears to be important in various areas of undergraduate and postgraduate medical education. But how can mindfulness, openness and a certain presence of mind, which are the conditions for serendipity, be used to make unexpected discoveries?

In recent years and decades, compulsory curricular structures have been established in order to ensure that the quality of undergraduate and postgraduate medical education is as uniform as possible and to provide learners with content-related orientation for the acquisition of knowledge, skills and attitudes. Serendipity, which can also be promoted and trained [7], was initially less at the center of activities. In the field of continuous professional development (CME), there is now even criticism that a focus on mandatory training activities could mean a loss of serendipity [8]. So perhaps it is time to take a look at whether serendipity could be integrated into undergraduate and postgraduate medical education as an important element of medical work. It is known from studies in non-medical fields that the targeted design of a campus [9] and working in heterogeneous teams combined with a culture of curiosity and experimentation can promote serendipity [10]. In medical studies, there are already interesting approaches in the realization of teaching buildings as well as in problem-oriented and interprofessional learning that can be used specifically to promote serendipity. Another possibility for supporting serendipity is learning how to collect information in an appropriate way [11]. In the medical field, this ability is relevant both for clinical thought processes and for scientific work. Changes of perspective and creativity exercises, such as those already used in undergraduate and postgraduate education with other learning objectives in the form of engaging with art [12], [13], are also suitable for promoting serendipity [14].

In this issue, Schwaab et al. [15] and Endres et al. [16] describe newly established elective courses. Such elective courses are presumably well suited for students with the appropriate curiosity to make unexpected discoveries. In a compulsory elective, as described by Scherg et al. [17], this could also be the case. Needs or motivational analyses of teachers or students, as presented in this issue by Hettkamp et al. [18], Rahn et al. [19], and de la Rosa et al. [20] can also be a first step in identifying opportunities where the unexpected can be perceived. This issue also contains other interesting topics on teaching in the various healthcare professions. At this point, the discovery of the unexpected is left to the serendipity of the interested reader.

## Author’s ORCID

Sigrid Harendza: [0000-0002-7920-8431]

## Competing interests

The author declares that she has no competing interests.
